# Undiagnosed Diabetes and Prediabetes in Yemen: A Growing Public Health Crisis in the Shadow of Conflict

**DOI:** 10.3390/medicina62010087

**Published:** 2025-12-31

**Authors:** Mohammed A. M. Y. Al-Hetar, Siti Liyana Saud Gany, Noradliyanti Rusli, Mohd Amir Kamaruzzaman, Wan Zurinah Wan Ngah, Shamsul Azhar Shah, Abdullah Almatary, Norasyikin A. Wahab

**Affiliations:** 1 Department of Medicine, Faculty of Medicine, Universiti Kebangsaan Malaysia, Jalan Yaacob Latif, Cheras, Kuala Lumpur 56000, Malaysia; p143260@siswa.ukm.edu.my; 2 Medical City Complex, The Specialised Clinic for Endocrinology and Diabetes, Ibb 6001196, Yemen; 3 Department of Biochemistry, Faculty of Medicine, Universiti Kebangsaan Malaysia, Jalan Yaacob Latif, Cheras, Kuala Lumpur 56000, Malaysia; liyanagany@ukm.edu.my (S.L.S.G.); wwanzurinah@yahoo.com (W.Z.W.N.); 4 Faculty of Bioeconomics, Food & Health Sciences, University Geomatika Malaysia, Taman Setiawangsa, Kuala Lumpur 54200, Malaysia; noradliyantirusli@geomatika.edu.my; 5 Department of Anatomy, Faculty of Medicine, Universiti Kebangsaan Malaysia, Jalan Yaacob Latif, Cheras, Kuala Lumpur 56000, Malaysia; mohdamir@ukm.edu.my; 6 Medical Innovation Research Centre, Shiga Universiti of Medical Sciences, Otsu 520-2192, Shiga, Japan; 7 Department of Community Health, Faculty of Medicine, Universiti Kebangsaan Malaysia, Jalan Yaacob Latif, Cheras, Kuala Lumpur 56000, Malaysia; drsham@hctm.ukm.edu.my; 8 Department of Surgery, Jiblah University for Medical and Health Sciences, Ibb 600196, Yemen; almatari95@gmail.com; 9 Department of Medicine, Hospital Canselor Tuanku Mukhriz, Jalan Yaacob Latif, Bandar Tun Razak, Cheras, Kuala Lumpur 56000, Malaysia

**Keywords:** undiagnosed diabetes, prediabetes, fasting blood sugar, OGTT, HbA1c

## Abstract

*Background and Objectives:* Type 2 diabetes mellitus (T2DM) is increasing in prevalence worldwide, placing a substantial burden on healthcare systems, particularly in resource-limited settings. In Yemen, limited screening and diagnostic capacity contribute to delayed detection and management. Prediabetes, a reversible state of dysglycemia, carries significant cardiovascular risk and frequently progresses to diabetes. Early identification of both conditions is vital for prevention and public health planning. *Materials and Methods:* This cross-sectional study, conducted from July 2024 to May 2025 in three medical centers in Ibb Governorate, Yemen, assessed 1045 adults aged 18–60 years without known diabetes or prediabetes. Glycaemic status was classified according to the 2025 American Diabetes Association criteria. Undiagnosed diabetes was defined using three diagnostic combinations: FBS + OGTT, FBS + HbA1c, and OGTT + HbA1c. *Results:* The prevalence of undiagnosed diabetes was 8.4% (FBS + OGTT) and 9.76% (FBS + HbA1c or OGTT + HbA1c). Prediabetes prevalence was 23.4%, 14.7% and 26.4% based on FBS, OGTT, and HbA1c, respectively. Females represented a higher proportion of undiagnosed diabetes and prediabetes cases. Age was significantly associated with glycemic status across all tests, while gender showed significant associations with FBS and HbA1c. Family history of chronic disease was significantly associated with HbA1c-based classification. Approximately 8–10% of adults in Ibb had undiagnosed diabetes, while up to one-quarter had prediabetes. Age and family history were key predictors of dysglycaemia. *Conclusions:* These findings highlight the need for targeted, multi-marker screening and early intervention strategies, particularly in relatively stable regions of conflict-affected settings, to prevent progression to diabetes and reduce long-term complications and healthcare burden.

## 1. Introduction

Diabetes mellitus (DM) is a chronic metabolic disorder characterised by persistent hyperglycemia resulting from impaired insulin secretion, insulin action, or both [1]. Globally, the burden of diabetes has reached critical levels, with an estimated 537 million adults affected in 2021, a figure projected to rise to 643 million by 2030 [2]. Type 2 diabetes mellitus (T2DM), which accounts for over 90% of cases, is strongly associated with ageing, sedentary lifestyles, and genetic predisposition [3]. These global trends are particularly concerning for low-resource and conflict-affected countries such as Yemen, where limited screening capacity and fragmented healthcare systems increase the risk of underdiagnosis and delayed management.

In low- and middle-income countries (LMICs), the diabetes burden is escalating disproportionately due to rapid urbanization, dietary transitions, and limited access to preventive care [4]. The Middle East and North Africa (MENA) region faces particularly steep increases in prevalence. Yemen is among the countries with scarce epidemiological data and a fragile public health infrastructure [5]. Surveillance systems remain fragmented, and regional disparities in disease burden are poorly documented [6].

According to the International Diabetes Federation (2025), the MENA region has one of the highest diabetes prevalence globally, with regional adult (20–79 years old) rates substantially above the global average (~11.1%) [7]. In neighbouring Saudi Arabia, national IDF data indicate adult diabetes prevalence exceeding 20%, with many cases undiagnosed [8]. These trends highlight the urgent need for scalable screening strategies and targeted public health interventions across the region, including Yemen.

Ibb Governorate, located in central Yemen, is densely populated and vulnerable to non-communicable diseases (NCDs) due to socioeconomic instability and prolonged conflict [9]. The use of multiple diagnostic tools, fasting blood glucose (FBS), oral glucose tolerance test (OGTT), and glycated haemoglobin (HbA1c), is essential for accurate classification and risk stratification [10]. Although HbA1c is widely adopted, its accuracy may be affected by hemoglobinopathies, iron-deficiency anaemia, and nutritional deficiencies, which are common in Yemen and other LMICs [11]. Anemia prevalence estimates in Yemen are substantial, where approximately 44.2% among pregnant women, 38% among adolescents, and over one-third of women of reproductive age, according to population data, indicating a significant public health burden that may influence HbA1c interpretation. These limitations underscore the importance of multimodality screening in conflict-affected populations.

Prediabetes represents an intermediate metabolic state characterized by impaired fasting glucose (IFG) and/or impaired glucose tolerance (IGT), conferring a high risk of progression to T2DM and related complications [12]. Globally, IGT estimated 9.1% (464 million adults) of adults in 2021 and is projected to rise to 10.0% (638 million) by 2045, while IFG prevalence is expected to increase from 5.8% (298 million) to6.5% (414 million) over the same period [13]. The burden of prediabetes is increasing most rapidly in low-income countries, where underdiagnosis remains common due to limited screening and laboratory access. In Yemen, early identification of prediabetes using combined glycaemic measures (FBS, OGTT, and HbA1c) provides a critical opportunity for intervention to prevent progression to overt diabetes, particularly in populations with high rates of undiagnosed dysglycaemia [14].

Demographic factors such as age and gender play a critical role in diabetes risk, with prevalence increasing markedly after age 40 [15]. Gender differences have also been observed in both biological susceptibility and health-seeking behaviour [16,17]. In Yemen, these demographic influences are further shaped by deeply rooted socioculturalnorms and ongoing conflict. Women often depend on male family members for permission or accompaniment to access medical services, and such restrictions on autonomy can delay disease recognition and management [18]. These sociocultural barriers are compounded by widespread health-system disruption resulting from prolonged conflict, leading to reduced access to basic healthcare services, including diabetes screening services [19]. Policy analyses further indicate that weak governance and fragmented service delivery discourage health-seeking behaviour and uptake of preventive services [20]. Additionally, recent evidence highlights low awareness of NCD risk factors and screening benefits among Yemeni adults, reflecting sociocultural and educational gaps that continue to hinder early detection efforts [21].

This study addresses a critical gap by estimating the prevalence of undiagnosed T2DM and prediabetes in Ibb Governorate using three diagnostic modalities (FBS, OGTT, HbA1c) and examining their associations with age and gender. The findings aim to inform region-specific screening strategies and contribute to the broader understanding of diabetes epidemiology in conflict-affected LMICs.

## 2. Materials and Methods

This cross-sectional study was conducted from July 2024 to May 2025 in Ibb Governorate, Yemen, across three major medical centers: Medical City Complex, Jiblah University for Medical and Health Sciences, and Al Noor Hospital. A convenience sampling approach was employed, targeting all employees of these centres and their family members or friends to ensure ease of communication and follow-up. Recruitment was facilitated through institutional networks and social media announcements.

**Figure 1:**
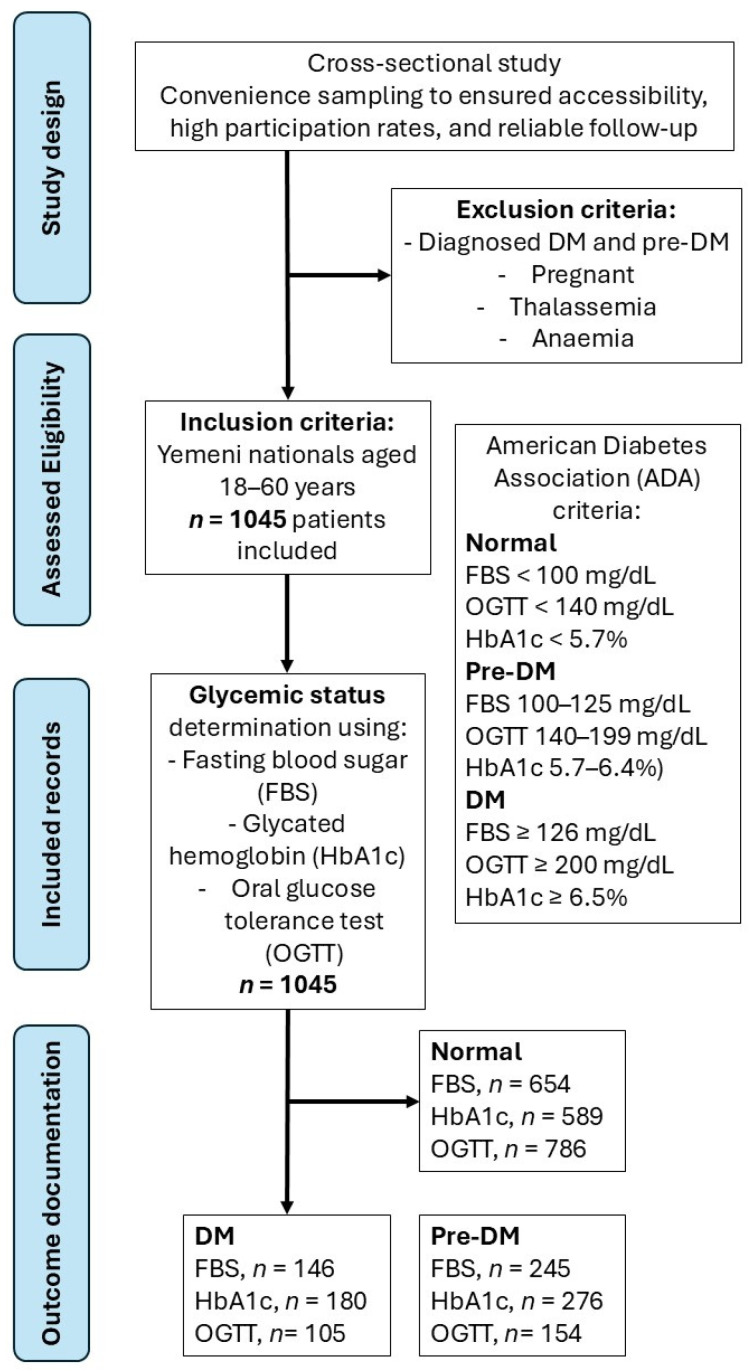
Study flowchart.

Eligible participants were Yemeni nationals aged 18–60 years who were non-diabetic or prediabetic and non-pregnant (as shown in Figure 1). Participants with known hemoglobinopathies (e.g., thalassemia) or clinically significant anaemia were excluded based on medical history and full blood count (FBC) results to minimize potential interference with HbA1c measurements. Ethical approval was obtained from the Research Ethics Committee, Universiti Kebangsaan Malaysia (UKM-Malaysia JEP-2024-680), and the Research Ethics Committees of Jiblah University (IRB Reference No. JiblahUNI.YEM.772756 and Registration No.: Jiblah UNI.YEM.2025.571). This study was funded by UKM (Grant GFFP: FF-2024-433).

Written informed consent was obtained from all participants following verbal and written explanations in Arabic, in accordance with institutional and national ethical standards and the Declaration of Helsinki (1964). Data collection included sociodemographic information, family history of diabetes and other chronic diseases, and biochemical measurements.

All participants fasted for at least 8 h before blood sampling. A 3 mL venous blood sample was collected to measure fasting blood sugar (FBS), glycated haemoglobin (HbA1c), and full blood count (FBC). FBS and 2 h post-prandial blood glucose (2hppBG) were analysed using an automated chemistry analyzer (Cobas 6000 series, Roche Diagnostic, Basel, Switzerland). HbA1c was measured using the Standard F HbA1c system (SD Biosensor, Suwon-si, Republic of Korea), employing standardized assays aligned with ADA recommendations. Full blood count analysis was performed using a 5-part differential automated hematology analyzer (Sysmex, Kobe, Japan). Subsequently, participants underwent an oral glucose tolerance test (OGTT) with ingestion of 75 g of glucose dissolved in 200 mL of water, and a second blood sample was collected 2 h post-ingestion for glucose measurement. All blood samples were analysed immediately after collection, in accordance with standard laboratory protocols, and no samples were stored before analysis.

Glycaemic status was classified according to the American Diabetes Association (ADA) criteria as normal (FBS < 100 mg/dL, OGTT < 140 mg/dL, HbA_1_c < 5.7%), prediabetes (FBS 100–125 mg/dL or OGTT 140–199 mg/dL or HbA_1_c 5.7–6.4%), or diabetes (FBS ≥ 126 mg/dL or OGTT ≥ 200 mg/dL or HbA_1_c ≥ 6.5%). Three diagnostic definitions of undiagnosed diabetes were applied in our analyses: (1) elevated FBS (≥126 mg/dL and OGTT (≥200 mg/dL); (2) elevated OGTT (≥200 mg/dL) and HbA1c (≥6.5%); and (3) elevated HbA1c (≥6.5%) and FBS (≥126 mg/dL).

Data were analysed using SPSS version 26 (IBM, Armonk, NY, USA). Normality of continuous variables was assessed using the Shapiro–Wilk test before analysis. Continuous variables with normal distribution were expressed as mean ± standard deviation (SD), while non-normally distributed variables were summarised as median and interquartile range (IQR). Associations between glycaemic status and predictor variables, including age, gender, family history, and biochemical results, were assessed using the chi-square test. The statistical significance was set at *p* < 0.05.

## 3. Results

### 3.1. Demographic and Clinical Characteristics

A total of 1045 participants were enrolled, comprising 428 males (40.95%) and 617 females (59.05%). The median age was 46 years (IQR: 35–55). The largest proportion of participants was in the 18–30-year age group (33.9%), followed by the 31–40-year age group (27.1%), the 41–50-year age group (22.5%), and the 51–60-year age group (16.6%). Approximately 41.7% (*n* = 257) of the participants were female, aged between 18 and 30 years.

Overall, 94.1% (983) of participants reported no family history of chronic diseases, and 95.5% of them were female. The most commonly reported family conditions were diabetes mellitus (2.7%, 28), hypothyroidism (1.3%, 14), hypertension (1.2%, 13), hypertension with diabetes (0.4%, 4), and cardiac disease (0.3%, 3), as shown in Table 1.

### 3.2. Glycaemic Status Distribution by Diagnostic Test

Glycemic classification was assessed using three diagnostic tests: fasting blood sugar (FBS), oral glucose tolerance test (OGTT), and HbA1c. The prevalence of prediabetes, based on FBS, OGTT, and HbA1c, was 245 (23.4%), 154 (14.7%), and 276 (26.4%), respectively. These results demonstrated variation in diagnostic yield across tests, with HbA1c identifying more prediabetic cases compared with FBS or OGTT (Table 2).

Across all age categories, females consistently represented a higher proportion of prediabetes participants, particularly in the 18–30- and 31–40-year age groups. In contrast, older age groups (41–60 years) showed higher rates in males. (Table 3).

### 3.3. Associations Between Participant Characteristics and Glycaemic Status

A significant association was found between age group and glycemic status across all tests: FBS (χ^2^: 141.27, *p* < 0.001), HbA1c (χ^2^: 251.90, *p* < 0.001), and OGTT (χ^2^: 184.25, *p* < 0.001). Gender was significantly associated with FBS (χ^2^: 10.50, *p* = 0.005) and HbA1c (χ^2^: 15.18, *p* = 0.001), but not with OGTT (χ^2^: 2.92, *p* = 0.232). A significant association was observed between family history of chronic disease and HbA1c classification (χ^2^: 21.11, *p* = 0.020). These findings suggest that HbA1c is more sensitive to underlying familial risk factors and gender-related metabolic differences (Table 4).

Post hoc adjusted residual analysis provided further insight into these associations. Younger adults (18–30 years) were much more frequently found in the normal glycaemic range, and far less frequently classified as prediabetic or diabetic across all tests. In contrast, individuals aged 41–60 years were more often classified as prediabetic or diabetic, and less frequently found in the normal category, indicating a clear age-related shift toward dysglycaemia. Gender differences also emerged in HbA1c and FBS results, where females were more commonly in the normal HbA1c category and less frequently in the prediabetes range, while males were more frequently classified as prediabetic in both HbA1c and FBS. Although family history showed a significant overall association with HbA1c status, no specific disease subgroup appeared more or less frequent than expected after Bonferroni correction, suggesting that the association was general rather than driven by any single condition (Table 5).

### 3.4. Prevalence of Undiagnosed Diabetes by Combined Diagnostic Criteria

The highest prevalence of undiagnosed diabetes was observed using FBS + HbA1c and OGTT + HbA1c, each detecting 102 cases (9.76%). In comparison, the FBS + OGTT combination identified 88 cases (8.42%). Across all combinations, females accounted for a slightly higher proportion of undiagnosed cases across all diagnostic combinations, as shown in Table 5. These results underscore the importance of using multiple diagnostic modalities to improve detection rates, particularly among females who may be underdiagnosed by single-test approaches (Table 6).

## 4. Discussion

The prevalence of undiagnosed T2DM in Ibb Governorate, based on abnormalities in at least two glycaemic parameters, ranged from 8.4% to 10.2%. This estimate is comparable to recent global and regional patterns of diabetes prevalence. Updated reports from the IDF indicate that diabetes continues to affect approximately one in ten adults globally [2]. In neighboring MENA countries, the burden is substantially higher. In Saudi Arabia, recent IDF estimates indicate that more than one-fifth of adults are affected by diabetes, reflecting one of the highest prevalence rates in the region [8]. Similarly, in Oman, contemporary national and international data suggest that diabetes affects approximately 12–18% of the adult population [22]. These regional trends provide important context for interpreting the prevalence of undiagnosed diabetes observed in the present study. In contrast, substantially higher diabetes prevalence has been reported in Gulf Cooperation Council countries, including Kuwait (~19–25%), Qatar (~20–25%), and the United Arab Emirates (~20–21%), which consistently rank among the countries with the highest diabetes burden globally according to the IDF [23]. These regional similarities likely reflect shared dietary transitions, general urbanization, and evolving metabolic risk profiles across the Arabian Peninsula [24,25].

Importantly, our study also revealed a substantial burden of prediabetes, affecting 23.4% by FBS, 14.7% by OGTT, and 26.4% by HbA_1_c. The variation in prediabetes prevalence across these tests highlights diagnostic inconsistency among glycemic markers. Hence, using a multi-marker screening protocol may help to improve diagnostic accuracy and ensure early detection of individuals at risk for diabetes.

Our findings are consistent with evidence from recent regional literature. A systematic review by Al-Hetar et al., eported that Vitamin D supplementation showed promising effects on insulin sensitivity and glycaemic control in prediabetic individuals across MENA-based RCTs [26]. These findings support the potential role of vitamin D in modulating metabolic risk.

The use of multiple diagnostic parameters, including FBS, OGTT, and HbA_1_c, enabled more accurate case identification and reduced the risk of underdiagnosis. In the context of Yemen’s constrained healthcare system, our findings demonstrate the combining FBS, OGTT and HbA1c improves detection of undiagnosed diabetes compared with reliance on a single test, supporting WHO recommendations for integrated screening strategies [27]. However, it should be acknowledged that OGTT, while diagnostically valuable, can pose practical challenges in many low-resource or unstable environments. Standard OGTT requires fasting, prolonged patient time commitment, laboratory infrastructure, trained personnel, and reliable sample handlings, which are factors than can limit its feasibility for large-scale screening outside well-equipped facilities. In such settings, alternative approaches or simplified diagnostics may be more practicable [28].

Comparative studies reinforce our findings, such as the Mexico City Diabetes Study, which reported that 45.3% of individuals with normal OGTT had HbA1c≥ 6.5%, suggesting that HbA1c captures chronic hyperglycaemia not reflected in transient glucose excursions [29]. Similarly, Açmaz et al., found a higher prevalence of dysglycaemia by HbA1c (43.1%) than by OGTT (30.9%) in obese patients, with poor agreement between the methods (κ = 0.326) [30]. Meanwhile, Nor Azian Abdul Murad et al., demonstrated a higher prevalence of both diabetes and prediabetes among Malaysian adults when using HbA1c compared to FPG, echoing our findings and highlighting the diagnostic value of HbA1c [31]. Their study focused on FPG and HbA_1_c, while we used OGTT as a third test, capturing cases that might otherwise be missed.

Although some studies in African and South Asian populations have questioned the reliability of HbA1c, particularly in settings where nutritional deficiencies, anaemia, and haemoglobinopathies are common [32], These challenges are especially pertinent in conflict-affected and resource-limited contexts such as Yemen, where health system disruption and micronutrient deficiencies remain prevalent [33]. Nevertheless, our cohort demonstrated improved concordance and case detection when HbA1c, alongside FBS and OGTT was incorporated, supporting its value when interpreted cautiously within a multi-marker screening approach. Age-related trends in our data revealed a marked increase in diabetes prevalence after age 50, a pattern consistent with epidemiological findings across Asia. Studies in South Asian populations have similarly shown that ageing is associated with declining β-cell function and earlier β-cell exhaustion [34,35], while Chinese cohorts report parallel increases in insulin resistance and β-cell dysfunction in middle-aged and older adults [36]. Together, these findings support the biologically plausible rise in dysglycaemia observed in older age groups. Gender differences were also evident, whereby men were more frequently identified using HbA1c, while women were more often classified through fasting-based measures. However, these findings should be interpreted cautiously, as the convenience sampling strategy and differential healthcare access may have influenced gender representation rather than reflecting true population-level disparities. These findings reflect a combination of biological susceptibility and healthcare access, as demonstrated in studies from Lebanon [37], Afghanistan [38], and Bangladesh [39].

The estimated prevalence of undiagnosed diabetes in our study was 9.8% and this aligns with the global pooled estimate of 10.1% reported by Guariguata et al., suggesting comparability rather than underestimation [40].

Although our sample was limited to government staff recruited through convenience sampling, which may restrict generalizability, the observed prevalence is comparable to international estimates. In addition, recruitment through employees of the participating centres and their family members or friends may have introduced selection bias and limited population diversity, potentially under-representing individuals from different socioeconomic backgrounds. Recruitment was confined to accessible urban and peri-urban settings due to substantial logistical constraints, including limited public transportation, infrastructural barriers, and the practical difficulty of reaching remote or rural populations, independent of ongoing conflict. This sampling approach may therefore under-represent populations in less accessible areas with different socioeconomic and health profiles. As a result, the findings may not be fully generalizable to the wider Yemeni population, particularly those residing in less accessible or underserved areas. This limitation is common in facility-based studies, highlighting the need for broader population-level screening [41]. Overall, our findings reinforce the importance of multi-parametric screening and suggest that HbA1c, when used in conjunction with OGTT or FBS, enhances diagnostic sensitivity. -This study further benefits from multi-center sampling across three hospitals, which strengthens generalizability, while stratified random sampling ensures balanced representation by age and gender. The use of a three-biomarker strategy aligned with ADA criteria supports both diagnostic accurace and international comparability. However, HbA1c interpretation can be influenced by hemoglobinopathies and nutritional anemia, which has been reported in some low- and middle-income settings. In this study, participants were screened during recruitment based on medical history, and individuals with a known diagnosis of thalassemia were excluded during the consent process. Full blood count testing was performed for all participants and the mean hemoglobin level in the study population exceeded 10 g/dL, indicating that clinically significant anemia was unlikely in the recruited cohort. These measures reduced the potential influence of hemoglobinopathies and nutritional deficiencies in HbA1c measurements, which were interpreted alongside fasting blood glucose and OGTT in accordance with international recommendations [10,42].

The findings of this study have important policy implications for diabetes screening and public health planning in conflict-affected settings. Although Yemen continues to experience prolonged conflict, not all regions are actively affected, and relatively stable governorates such as Ibb retain functional healthcare facilities capable of implementing preventive strategies. The high prevalence of undiagnosed diabetes identified in this study highlights the need for targeted, facility-based screening programs in these stable areas, particularly for adults aged ≥ 40 years and those with a family history of chronic disease. Importantly, the substantial burden of prediabetes observed provides a critical opportunity for early intervention, as untreated prediabetes frequently progresses to overt diabetes and subsequent complications. From a health-system perspective, prioritizing early detection and prevention in stable regions may reduce long-term healthcare expenditures associated with advanced diabetes complications, allowing limited national resources to be allocated more efficiently toward broader health system strengthening and post-conflict recovery. These findings support the integration of multi-marker glycaemic screening into routine care in accessible regions as a pragmatic and cost-effective public health strategy in fragile and conflict-affected settings.

## 5. Conclusions

This study provides the first population-based estimate of undiagnosed T2DM in Ibb governorate, Yemen, confirming a prevalence of approximately 9.8% using combined glycaemic criteria, with prediabetes affecting up to one-quarter of adults. The findings demonstrated marked age-related increases in dysglycaemia, particularly after the age of 50, and highlight gender-specific diagnostic patterns, underscoring the importance of multi-marker screening approaches. From a public health perspective, there results support the implementation of targeted, facility-based multi-marker screening programmed in relatively stable regions of Yemen, with prioritization of adults aged ≥ 40 years and individuals with a family history of chronic disease. Integrating early detection and prediabetes management into existing primary healthcare services could help delay disease progression, reduce long-term complications, and alleviate future healthcare burden. Such strategies are particularly relevant for conflict-affected settings, where efficient use of limited resources is essential.

## Figures and Tables

**Table 1:** Demographic and clinical characteristics of participants.

Parameter	Total (*n*, %)	Male (*n*, %)	Female (*n*, %)
Total Participants	1045 (100%)	428 (40.95%)	617 (59.05%)
Age (years)	Median: 46 (IQR 35–55)		
*Age Group*			
18–30 years	354 (33.9%)	97 (22.7%)	257 (41.7%)
31–40 years	283 (27.1%)	126 (29.4%)	157 (25.4%)
41–50 years	235 (22.5%)	110 (25.7%)	125 (20.3%)
51–60 years	173 (16.6%)	95 (22.2%)	78 (12.6%)
*Family History of Chronic Disease*			
None	983 (94.1%)	394 (92.1%)	589 (95.5%)
Diabetes Mellitus	28 (2.7%)	19 (4.4%)	9 (1.5%)
Hypothyroidism	14 (1.3%)	5 (1.2%)	9 (1.5%)
Hypertension	13 (1.2%)	9 (2.1%)	4 (0.6%)
Hypertension and Diabetes	4 (0.4%)	1 (0.2%)	3 (0.5%)
Cardiac Disease	3 (0.3%)	3 (0.7%)	0 (0.0%)

Legend: Demographic and clinical characteristics of the study participants (*n* = 1045), including age distribution, gender composition, and self-reported family history of chronic diseases. Age is presented as median with interquartile range (IQR), and categorical variables are expressed as number (*n*) and percentage (%). Percentages for male and female participants are calculated within each category.

**Table 2:** Prevalence of prediabetes based on diagnostic tests.

Diagnostic Test	Prediabetes (*n*, %)	Male (*n*, %)	Female (*n*, %)	*p*-Value
Fasting Blood Glucose (FBS)	245 (23.4%)	122 (28.50%)	123 (19.94%)	<0.001
Oral Glucose Tolerance Test (OGTT)	154 (14.7%)	72 (16.82%)	82 (13.29%)	0.1132
Glycated Hemoglobin (HbA1c)	276 (26.4%)	138 (32.24%)	138 (22.37%)	<0.001

Legend: Prevalence of prediabetes among study participants (*n* = 1045) based on FBS, OGTT and HbA1c. Data are presented as number (*n*) and percentage (%). Male and female counts represent the distribution within each diagnostic category. *p*-values refer to comparisons of prediabetes prevalence between genders for each diagnostic test using the chi-square test.

**Table 3:** Prediabetes status by age group and gender.

Age Group	Test	Male (*n*, %)	Female (*n*, %)
18–30 years old	FBS	12 (25.0%)	36 (75.0%)
	OGTT	3 (20.0%)	12 (80.0%)
	HbA1c	14 (31.1%)	31 (68.9%)
31–40 years old	FBS	35 (46.7%)	40 (53.3%)
	OGTT	16 (40.0%)	24 (60.0%)
	HbA1c	44 (46.3%)	51 (53.7%)
41–50 years old	FBS	47 (61.0%)	30 (39.0%)
	OGTT	25 (49.0%)	26 (51.0%)
	HbA1c	45 (54.9%)	37 (45.1%)
51–60 years old	FBS	28 (62.2%)	17 (37.8%)
	OGTT	28 (58.3%)	20 (41.7%)
	HbA1c	35 (64.8%)	19 (35.2%)

Legend: Distribution of prediabetes cases by age group and gender according to FBS, OGTT and HbA1c. Data are presented as number (*n*) and percentage (%), with percentages calculated within each age groups and diagnostic test.

**Table 4:** The association between glycaemic indices and demographic data.

Variable Pair		Normal (N)	Prediabetes (N)	Diabetes (N)	χ^2^(df)	*p*-Value	Cramer’s V (Significance)
*Gender X Glycaemic Status*							
OGTT	Female	475	82	60	2.924 (2)	0.232	0.053
	Male	311	72	45			(0.232)
HbA1c	Female	376	138	103	15.18 (2)	<0.001 *	0.121
	Male	213	138	77			(0.001 *)
FBS	Female	406	123	88	10.5 (2)	<0.001 *	0.100
	Male	248	122	58			(0.005 *)
*Age X Glycaemic Status*							
OGTT	18–30 y/o	335	15	4	184.25 (6)	<0.00 *	
	31–40 y/o	226	40	17			0.297
	41–50 y/o	145	51	39			(<0.001 *)
	51–60 y/o	80	48	45			
HbA1c	18–30 y/o	300	45	9	251.9 (6)	<0.001 *	
	31–40 y/o	154	95	34			0.347
	41–50 y/o	87	82	66			(<0.001 *)
	51–60 y/o	48	54	71			
FBS	18–30 y/o	295	48	11	141.27 (6)	<0.001 *	
	31–40 y/o	176	75	32			0.26
	41–50 y/o	105	77	53			(<0.001 *)
	51–60 y/o	78	45	50			
*Family History X Glycaemic Status*							
OGTT	NAD	743	143	97	8.45 (10)	0.585	
	DM	18	5	5			
	Hypothyroidism	11	1	2			0.064
	HTN	10	2	1			(0.585)
	HTN + DM	2	2	0			
	Cardiac Disease	2	1	0			
HbA1c	NAD	564	250	169	21.11 (10)	0.02 *	
	DM	9	12	7			
	Hypothyroidism	9	2	3			0.101
	HTN	5	7	1			(0.02 *)
	HTN + DM	1	3	0			
	Cardiac Disease	1	2	0			
FBS	NAD	620	227	136	6.95 (10)	0.73	
	DM	14	9	5			
	Hypothyroidism	9	2	3			0.058
	HTN	8	4	1			(0.73)
	HTN + DM	2	2	0			
	Cardiac Disease	1	1	1			

Legend: Association between glycaemic status (normal, prediabetes and diabetes) and demographic and clinical variables (gender, age group, and family history of chronic disease) based on FBS, OGTT and HbA1c > Data are presented as counts (N). Associations were assessed using the chi-square (χ^2^) test, with degrees of freedom (df) indicated. Effect size was estimated using Cramer’s V. *p*-values < 0.05 were considered statistically significant and are marked with an asterisk (*). Abbreviations: NAD, no associated disease; DM, diabetes mellitus; HTN, hypertension.

**Table 5:** Adjust residuals (AR) and Bonferroni-corrected *p*-values between glycaemic indices and demographic data.

Variable Pair		Normal AR	Bonferroni *p*	Prediabetes AR	Bonferroni *p*	Diabetes AR	Bonferroni *p*
*Gender X Glycaemic Status*							
OGTT	Female	1.591	0.669	−1.584	0.679	−0.417	1.000
	Male	−1.591	0.669	1.584	0.679	0.417	1.000
HbA1c	Female	3.582	0.002 *	−3.561	0.002 *	−0.546	1.000
	Male	−3.582	0.002 *	3.561	0.002 *	0.546	1.000
FBS	Female	2.582	0.059	−3.216	0.008 *	0.326	1.000
	Male	−2.582	0.059	3.216	0.008 *	−0.326	1.000
*Age X Glycaemic Status*							
OGTT	18–30 y/o	10.406	<0.001 *	−6.853	<0.001 *	35.57	<0.001 *
	31–40 y/o	2.119	0.409	−0.335	1.000	28.44	0.097
	41–50 y/o	−5.45	<0.001 *	3.421	0.008 *	23.61	0.002 *
	51–60 y/o	−9.662	<0.001 *	5.284	<0.001 *	17.38	<0.001 *
HbA1c	18–30 y/o	13.242	<0.001 *	−7.19	<0.001 *	−8.997	<0.001 *
	31–40 y/o	−0.773	1.000	3.198	0.017 *	−2.719	0.079
	41–50 y/o	−6.791	<0.001 *	3.35	0.01 *	5.008	<0.001 *
	51–60 y/o	−8.309	<0.001 *	1.568	1.000	9.081	<0.001 *
FBS	18–30 y/o	9.921	<0.001 *	−5.399	<0.001 *	−7.251	<0.001 *
	31–40 y/o	−0.16	1.000	1.421	1.000	−1.514	<0.001 *
	41–50 y/o	−6.442	<0.001 *	3.831	0.002 *	4.31	<0.001 *
	51–60 y/o	−5.206	<0.001 *	0.872	1.000	6.201	<0.001 *
*Family History X Glycaemic Status*							
OGTT	NAD	1.102	1.000	−0.688	1.000	−0.771	1.000
	DM	−1.358	1.000	0.472	1.000	1.393	1.000
	Hypothyroidism	0.293	1.000	−0.807	1.000	0.531	1.000
	HTN	0.144	1.000	0.066	1.000	−0.284	1.000
	HTN + DM	−1.17	1.000	1.993	0.832	−0.67	1.000
	Cardiac Disease	−0.343	1.000	0.91	1.000	−0.58	1.000
HbA1c	NAD	2.626	0.156	−2.859	0.077	−0.111	1.000
	DM	−2.62	0.158	2.001	0.817	1.104	1.000
	Hypothyroidism	0.602	1.000	−1.036	1.000	0.419	1.000
	HTN	−1.31	1.000	2.258	0.431	−0.916	1.000
	HTN + DM	−1.267	1.000	2.208	0.490	−0.914	1.000
	Cardiac Disease	−0.805	1.000	1.584	1.000	−0.791	1.000
FBS	NAD	1.299	1.000	−1.071	1.000	−0.505	1.000
	DM	−1.395	1.000	1.101	1.000	0.601	1.000
	Hypothyroidism	0.132	1.000	−0.814	1.000	0.81	1.000
	HTN	−0.078	1.000	0.627	1.000	−0.657	1.000
	HTN + DM	−0.521	1.000	1.256	1.000	−0.808	1.000
	Cardiac Disease	−1.048	1.000	0.405	1.000	0.969	1.000

Legend: Post hoc analysis of associations between glycaemic status (normal, prediabetes, and diabetes) and participants’ characteristics (gender, age groups, and family history of chronic disease) using adjusted residuals (AR) with Bonferroni-corrected *p*-values for FBS, OGTT, and HbA1c. Adjusted residuals indicate the strength and direction of deviation from expected frequencies within each category. Bonferroni-corrected *p*-values < 0.05 were considered statistically significant and are marked with an asterisk (*). Abbreviations: NAD, no associated disease; DM, diabetes mellitus; HTN, hypertension.

**Table 6:** Prevalence of undiagnosed diabetes by combined diagnostic criteria.

Diagnostic Test Pair	Undiagnosed Diabetes (*n*, %)	Male (*n*, %)	Female (*n*, %)
FBS + OGTT	88 (8.42%)	37 (3.54%)	51 (4.88%)
FBS + HbA1c	102 (9.76%)	43 (4.11%)	59 (5.64%)
OGTT + HbA1c	102 (9.76%)	43 (4.11%)	59 (5.64%)

Legend: Prevalence of undiagnosed diabetes based on paired diagnostic criteria using FBS, OGTT, and HbA1c. Data are presented as number (*n*) and percentages (%), with percentages calculated relative to the total study population (*n* = 1045). Male and female values indicate gender-specific contributions to the overall prevalence. *p*-values refer to comparisons between genders for each diagnostic test pair.

## Data Availability

The data presented in this study are available on request from the corresponding author.

## Abbreviations

The following abbreviations are used in this manuscript:

DA	American Diabetes Association
BMI	Body Mass Index
DM	Diabetes Mellitus
ER	Endoplasmic Reticulum
FBC	Full Blood Count
FBS	Fasting Blood Sugar/Fasting Blood Glucose
FPG	Fasting Plasma Glucose
HbA1c	Glycated Hemoglobin
HTN	Hypertension
IFG	Impaired Fasting Glucose
IGT	Impaired Glucose Tolerance
IQR	Interquartile Range
LMICs	Low- and Middle-Income Countries
MENA	Middle East and North Africa
NCDs	Non-Communicable Diseases
OGTT	Oral Glucose Tolerance Test
RCTs	Randomized Controlled Trials
SD	Standard Deviation
SPSS	Statistical Package for the Social Sciences
T2DM	Type 2 Diabetes Mellitus
UKM	Universiti Kebangsaan Malaysia
WHO	World Health Organization
